# Multi-objective optimal water resources allocation in the middle and upper reaches of the Huaihe River Basin (China) based on equilibrium theory

**DOI:** 10.1038/s41598-022-10599-w

**Published:** 2022-04-22

**Authors:** Zengchuan Dong, Jitao Zhang, Ke Zhang, Xinkui Wang, Tian Chen

**Affiliations:** 1 College of Hydrology and Water Resources, Hohai University, Nanjing, 210000 China; 2 International Institute for Infrastructural, Hydraulic and Environmental Engineering, Delft, 2628 AX The Netherlands

**Keywords:** Environmental social sciences, Hydrology

## Abstract

In the river basin water resources allocation (WRA) problem, an unbalanced WRA poses challenges to water resources management departments. Many studies focus on achieving a lower water shortage rate while ignoring the equilibrium relationship among the socio-economic system, water resources system and eco-environmental system, as well as the equilibrium relationship among different regions. In this study, a water resources allocation model(WRAM) based on equilibrium theory is constructed to achieve the balance between different systems and different spaces in a basin. First, the relationship among the water resources system, socio-economic system and eco-environmental system is described. Then, the regional equilibrium index and system equilibrium index are constructed. Finally, the first model based on equilibrium theory is constructed. The results show that: (1) the Pareto Front reflects the contradictory relationship between economic development and environmental sustainability; (2) with the restructuring of industry and cropping, both economic efficiency and water shortage rates improve; (3) the equilibrium of the basin could also be further improved if water resources utilisation is further improved. Therefore, this study improves the existing WRAM, which can be applied to guide the water resources management of river basin.

## Introduction

Water resources (WR) are considered to be an essential natural resource for human survival and development, and they support the circular development of an ecological environment system as the ecological resource^1,2^. With the rapid socio-economic development of China and the uncertainty of WR^3,4^, the conflict WR supply and demand has become increasingly prominent^5^. Water resources allocation (WRA) can reduce conflict through engineering measures and non-engineering measures, making the study of WRA a hot issue^6^. A water resources allocation scheme (WRAS) should not only pursue water consumption efficiency but also consider the equity and sustainability of WRA between the middle and upper reaches of a basin and between different water-using sectors. Therefore, how to allocate limited WR to water-using sectors and regions with competing relationships to realize a balanced state between different systems and spaces is the key to solving this problem.

In the past few decades, many studies on the efficiency of WRA have been conducted^7–11^. From a socio-economic perspective, the highest efficiency is achieved when limited water resources are available to maximize the satisfaction of economic development needs and human needs^12,13^. Xu et al. considered efficiency by calculating economic benefits^14^. Tian et al. took the amount of water shortage as the objective function and considered climate conditions as a variable^2^. These methodologies have been proven to be acceptable and appropriate, but current research on WRA is still primarily focused on economic benefits while neglecting equity and sustainability.

Regarding equity and sustainability, equity refers to equal access to WR across regions and water-using sectors within a basin, and sustainability means that the ecology of a basin is not damaged. Currently there are few studies on the equity and sustainability of WRA. Wang and Plazzo evaluated the equity performance of sponge city construction to enhance the understanding of the impact of stormwater management on communities^15^. The contemporary water resources allocation model (WRAM) mainly takes the satisfaction of the minimum eco-environmental water demand as a criterion for sustainability^16^. However, these studies of equity and sustainability are insufficiently comprehensive and they neglect the connections between the water systems within regions. The conflict between water-using systems and water-using space still constitutes an obstacle to WRA.

To fill the gap in this field, this study attempts to explore options for realizing equitable, efficient and sustainable water resources management (WRM). This study constructs a multi-objective optimal water resource allocation model based on equilibrium theory to achieve coordination and stability between water-using sectors and equitable and sustainable development between regions. In the model, efficiency is set as an objective function, which maximizes the satisfaction of water needs for human life and economic development. Equity is set as a constraint and this study adopts the Gini coefficient, which is often applied to assess income equity, to measure the equity of WRA. For sustainability, this study considers the minimum of typical pollutant emissions as an objective function and the coupling coordination degree (CCD) between water-using systems as a constraint, on the basis of fully meeting ecological water demand.

At present, WRA methods are increasingly comprehensive, but research on balance in WRA is still in the development stage. Syme et al. thought that it is important that the balance be considered in WRA decision-making^17^. Balance in WRA mainly includes two parts: the balance between different spaces in a basin and the balance between the socio-economic system and eco-environment system in the space. This is the concept of WRA equilibrium. Equilibrium should be included as an important concept in WRM system. If the balance is not considered, an unreasonable WRAS may lead to risks in a certain region in a basin or a certain system in a region.

Shu and Xiong evaluated the balance of regional development in China^18^. For system equilibrium, Fang et al. and Guan et al. revealed the equilibrium relationship between various systems at the urban city level through a system dynamics model^19,20^. Wang and Li combined the logarithm mean Divisia index (LMDI) model with the Tapio model to establish model for evaluating the relationship between the water resources system and the economic system^21^. Although equilibrium research has been conducted in other fields, there is little research on equilibrium in WRA, and there is no unified method or theory to mitigate the conflicts between different regions and different systems.

Therefore, the objectives of this paper are: (1) to define the meaning of WRA equilibrium; (2) to propose a measure index of regional equilibrium and system equilibrium; (3) to establish an optimal WRA model on the basis equilibrium theory; and (4) to quantify the optimal WRAM and propose a reasonable WRAS. By meeting these goals, this paper will provide a new method of WRA and make the WRAM more perfect and more applicable. The remainder of this paper is structured as follows. The “Methodology and Materials” section introduces the method of equilibrium theory and the basic situation of the Huaihe River Basin in China, followed by the content of optimal WRAM in “Modelling” section. Then, the results of WRA under various scenarios are analyzed in “Results” section. At last, a wide range of conclusions and some consideration to the possibility of future research are summarized in “Conclusions” section.

## Methodology

**Figure 1:**
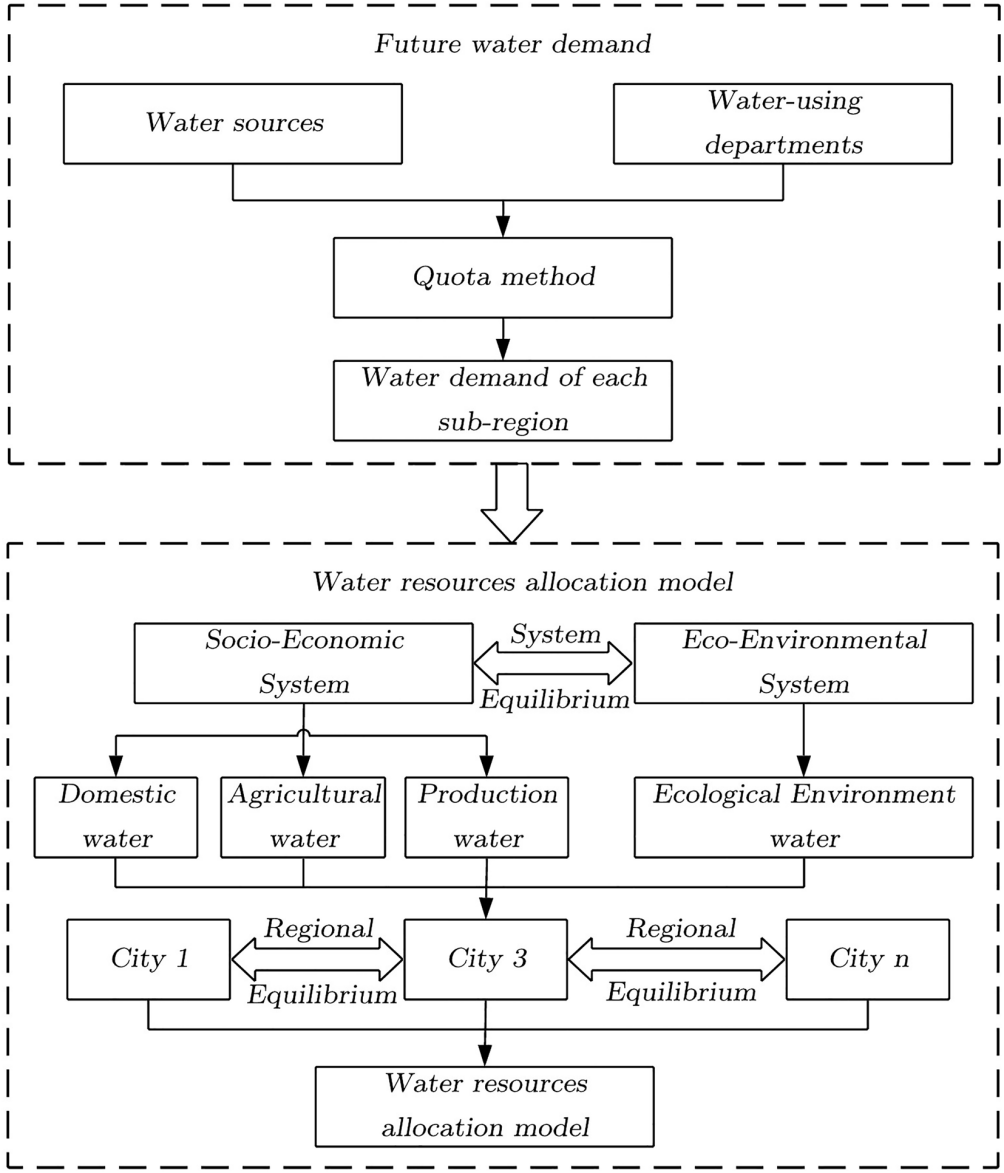
Flowchart of the methodology.

Because river basin management devotes too much attention to the efficiency of WRA while neglecting equity and sustainability, this study constructs a multi-objective optimal WRAM that includes efficiency, equity and sustainability based on equilibrium theory. This study takes the following three steps: (1) interpreting regional and system equilibria, (2) forecasting water demand in the water-using sector based on the quota method, and (3) constructing an equilibrium theory-based optimal WRAM to optimize the allocation of water resources under different scenarios. The flowchart of the proposed method is shown in Fig. 1.

### Interpretation of regional equilibrium and system equilibrium

In economics, the term “equilibrium” refers to a relatively static and stable state achieved by variables related to economic matters under certain conditions. To date, some scholars have considered equilibrium in other fields. Wang and Ducret evaluated the regional equilibrium of China's traffic arteries^22^. Nivala et al. proposed a GIS-based method to estimate the regional balance of forest resources^23^. There have been few studies of equilibrium theory in the field of water resources. As a common-pool and unidirectional natural resource, WR involves many regions and systems^24^. Consequently, there is a greater requirement for a well-balanced allocation of water resources. This study believes that equilibrium is an expression of equity. Therefore, based on equilibrium theory, this study divides equilibrium into regional equilibrium and system equilibrium. Regional equilibrium refers to the fact that when there are different regions within a watershed competing for the same natural resources, the development of one side should not neglect the demand of another side. System equilibrium implies that watershed authorities should ensure the coordination, stability and balance between the socio-economic system and the ecological system.

In economics, the Gini coefficient can be taken as a measure of the equilibrium of income distribution^25^. Some scholars have applied it to other fields. Cullis and Koppen applied the Gini coefficient to measure the balance of land resource distribution^26^. In systems theory, the CCD is adopted to indicate the equilibrium state between systems. Zammer et al. investigated the CCD between natural resources and financial development based on natural resources and socio-economic data from western, central and eastern China, and they explored the coupling coordination state between the socio-economic system and the ecological system ^27^. According to studies in other fields, this study applies the Gini coefficient and the CCD to the field of WRM, and adopts the comprehensive Gini coefficient and CCD to measure the regional equilibrium and system equilibrium of WRA.

### Scenario setting

This study examines the WRA in 2021 and 2050 at a guaranteed rate of 75%. 2021 and 2050 are defined as typical years and are regarded as Scenarios 1 and 2, respectively. Compared to Scenario 1, the socio-economic development in Scenario 2 will be improved to varying degrees to show the characteristics of social development and the sustainability of the WRAM. In addition, a 10% improvement in water use efficiency is presented as Scenario 3 to analyse the impact of water use efficiency on WRA in the study area.

### Study area

**Figure 2:**
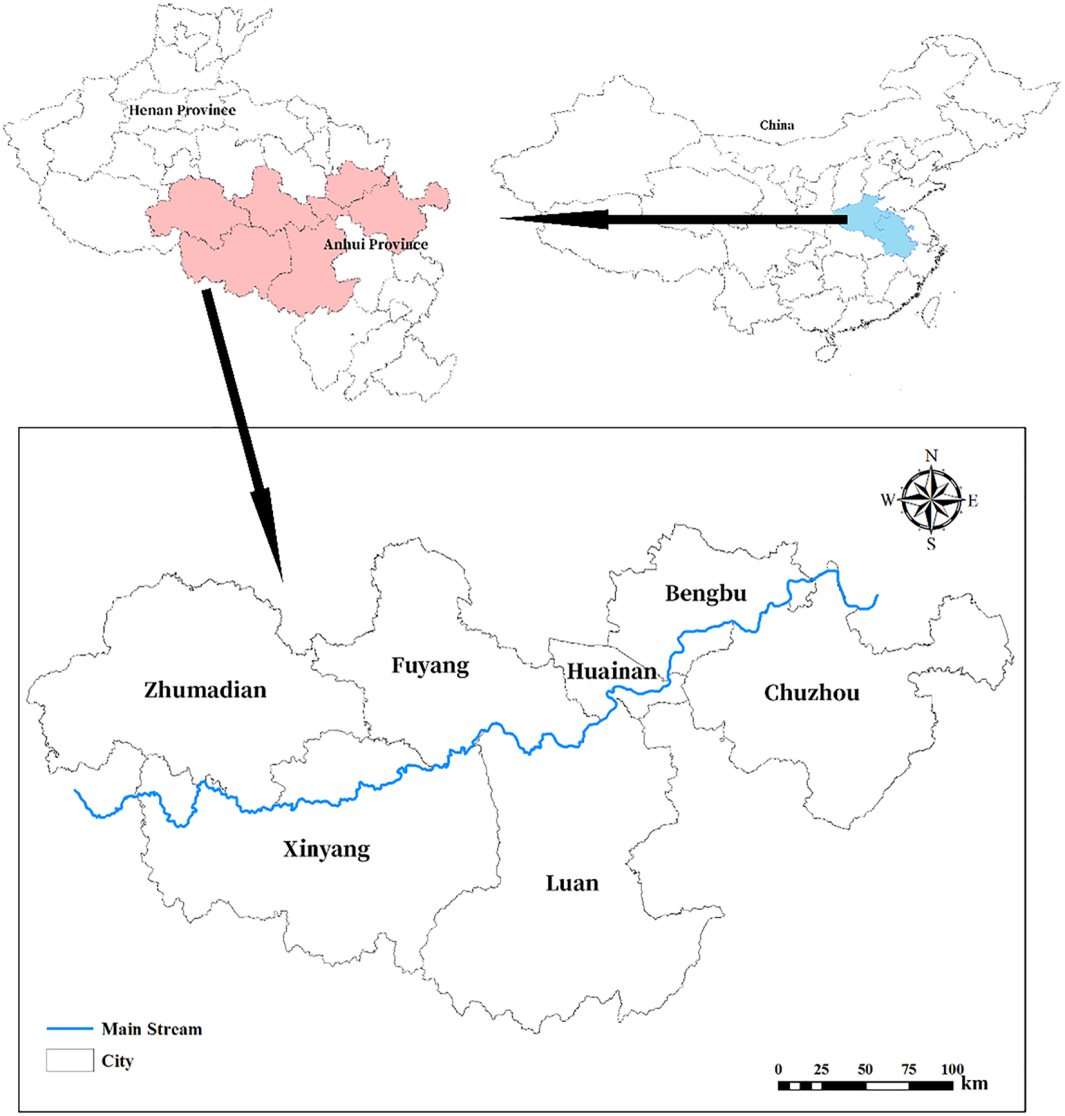
Location of the study area.

As shown in Fig. 2 (created by ArcMap 10.7 https://www.esri.com/en-us/arcgis/products/arcgis-desktop/resources), the middle and upper reaches of the mainstream of the Huaihe River Basin are located in eastern of China, a transitional climate zone between a warm temperate zone and a subtropical zone, with distinct seasonal monsoon climate characteristics^28^. The annual average temperature of the Huaihe River Basin is 15.3 ℃ and the average annual precipitation is 920 mm^29^. The study area spans two provinces, Henan and Anhui. It is densely populated, rich in land, rich in resources and convenient for transportation. As an important food production base, energy and mineral base, and manufacturing base in China, it plays a very important role in the overall economic and social development of China. In this study, seven prefecture-level cities with similar social and economic development level are selected as sub-regions based on administrative divisions^30^.

There are four kinds of water sources in the study area: surface water, groundwater, unconventional water and water supplied by other basins. The water supplied by other basins is mainly through the South to North Water Diversion Project Yellow River water transfer, and Yangtze River water transfer. The water consumption departments in the study area mainly include the agricultural water consumption department, domestic water consumption department, production water(secondary and tertiary industry) consumption department, and ecological environment water consumption department. The unilateral water resources production value coefficient of each water-using sector is shown in Table 1.

**Table 1:** Economic benefit coefficient of each water consumption department (CNY/m^3^).

	Domestic water	Agricultural water	Production water	Ecological and environment water
2021	342	26	159	200
2050	360	55	200	220

In this study, the socio-economic data and hydrological data are from the Huaihe River Basin Water Resources Bulletin (source: http://www.hrc.gov.cn/main/szygb/21448.jhtml), and the statistical yearbooks published by the Anhui provincial government, Henan provincial government and National Bureau of Statistics of China (source: http://tjj.ah.gov.cn/ssah/qwfbjd/tjnj/index.html, http://www.ha.stats.gov.cn/tjfw/tjcbw/tjnj/, http://www.stats.gov.cn/tjsj/ndsj/).

## Modelling

### Model construction

Before the introduction of the model, the following assumptions are made: (1) The study area is a single watershed, and only one watershed provides water for each sub-area. (2) The watershed management department plays a leading role, and fully understands the objective function and constraints of the allocation model. (3) There are no water right transactions among the districts in the basin. 4. Four water-using sectors are considered in the WRAM. 5. Three water supply sources are considered in the model.

### Objective function

Objective 1 (Maximization of Efficiency): Based on the principle of balance, efficiency, and equity, the maximum economic benefits is taken as the goal^31^:

1 $$ \max f_{1} (Q) = \sum\limits_{i = 1,j = 1,k = 1,t = 1}^{I,J,K,T} {b_{ijk} Q_{ijkt} } $$

In the formula, $i$ denotes the count of sub-regions, $t$ represents the count of period, $k$ is the count of water consumption departments and $j$ represents the count of water sources. $b_{ijk}$ is the unilateral water resources production value coefficient ($CNY/{\text{m}}^{{3}}$) of the water resources supplied from the source $j$ to the water use sector $k$ in the sub-region $i$. $Q_{ijkt}$ is the water resources quantity (m^3^) allocated by the source $j$ to water consumption department $k$ in the sub-regions $i$ in period $t$.

Objective 2 (Maximization of Sustainability): On the basis of sustainability development, take the minimum sum of Chemical Oxygen Demand (COD) emission in the basin as the objective^31^:

2 $$ \min f_{2} (Q) = \sum\limits_{i = 1,k = 1}^{I,K} {d_{ik} p_{ik} \sum\limits_{j = 1}^{J} {Q_{ijk} } } $$

where $d_{ik}$ represents the discharge of COD in unit sewage of water use department $k$ in sub-region $i$ (ton/m^3^) and $p_{ik}$ represents the coefficient of the sewage discharge of water consumption department $k$ in sub-region $i$.

### Constraint setting

#### Constraint 1

The water resources allocated from each water source to each sub-regions cannot exceed the capacity of each water source^31^.

3 $$ \sum\limits_{i = 1}^{I} {\sum\limits_{k = 1}^{K} {Q_{ijk} } } \le WR_{j} $$

where $WR_{j}$ represents the available water resources quantity of water source $j$.

#### Constraint 2

The water resources quantity allocated to each water use department in each sub-region cannot exceed the water requirement quantity of water use departments in each sub-area^31^.

4 $$ \sum\limits_{j = 1}^{J} {Q_{ijk} } \le R_{ik} $$

where $R_{ik}$ denotes the water requirement quantity of water use sector $k$ in the sub-region $i$.

#### Constraint 3

Regarding the concept of water allocation based on "regional equilibrium", the basic condition for the allocation of water resources to different sub-regions within a basin is that it is in accordance with the actual situation of regional social and economic development. If too many or too few water resources are given to a particular region, the balance between the various regions within the basin will be disrupted. Therefore, the combined Gini coefficient was chosen as the constraint for this study.

5 $$ Gini_{1} = 1 - \sum\limits_{n = 1}^{N} {(Q_{n} - Q_{n - 1} )(P_{n} + P_{n - 1} )} $$

6 $$ Gini_{2} = 1 - \sum\limits_{n = 1}^{N} {(Q_{n} - Q_{n - 1} )(G_{n} + G_{n - 1} )} $$

7 $$ \frac{1}{2}Gini_{1} + \frac{1}{2}Gini_{2} \le 0.5 $$

In the formula, $Gini_{1}$ is the Gini coefficient of water supply and population, $Gini_{2}$ is the Gini coefficient of water supply and GDP, $Q_{n}$ is the cumulative percentage of water supply, $P_{n}$ is the cumulative percentage of population, $G_{n}$ is the cumulative percentage of GDP, and $N$ stands for the number of cities. This paper considers that population and GDP are equally important, so the weight of $Gini_{1}$ and $Gini_{2}$ is 1/2.

#### Constraint 4

According to the equilibrium theory of water resources allocation described above, per capita GDP is selected as the evaluation index of coordinated development of the socio-economic subsystem in the study area, and COD emissions per 10 thousand (CNY) GDP are selected as the evaluation index of coordinated development of eco-environment subsystem in the study area.

The degree of coupling coordination is often taken as a measure of the coordination relationship between systems^32–34^. To meet the system equilibrium constraint, the coordination degree of socio-economic system and eco-environmental system should be controlled within a certain limit to ensure the stable development and equilibrium of the whole basin water resources socio-economic eco-environmental system. According to the research of other scholars, the CCD can be divided into four levels. If the CCD between subsystems is greater than 0.7, the system can be considered to be coordinated, and a balance between subsystems can be achieved^32–36^.

8 $$ E = \sqrt {C*T} \ge 0.7 $$

9 $$ C = \left[ {\frac{{I_{1} \cdot I_{2} }}{{\left[ {\left( {I_{1} + I_{2} } \right)/2} \right]^{2} }}} \right]^{\frac{1}{2}} $$

10 $$ T = \alpha I_{1} *\beta I_{2} $$

where $I_{1}$ is the evaluation index of the socio-economic subsystem and is a positive index. $I_{2}$ is the evaluation index of the ecological environment subsystem and is a negative index. Before calculation, $I_{1}$ and $I_{2}$ should be normalized. $C$ refers to the coupling degree between the socio-economic system and the eco-environmental system; $T$ is the evaluation index for the coordinated development between subsystems; $E$ is the coupling coordination degree; and $\alpha$ and $\beta$ represent the contributions of the subsystems.

### Solution

The NSGA-2^37^ algorithm is used to solve the WRAM problem. NSGA-2 is an evolutionary algorithm for solving optimization problems^38–40^. The algorithm uses a non-dominant sorting method to design the fittings. The non-dominated sequencing solution can be considered the equivalent solution of the Pareto frontier. The NSGA2 algorithm reduces the complexity of traditional genetic algorithms and has proven to be an appropriate algorithm for solving water resource management problems^41–43^.

The specific settings of the NSGA-2 algorithm parameters in this study are shown in Table 2.

**Table 2:** Specific parameter values of NSGA-2.

Generation	Population size	Crossover probability	Mutation probability
4000	100	0.9	0.1

### Performance of the algorithms

Because there are multiple sets of non-inferior solution sets for different parameter choices, two metrics are adopted in this study to evaluate the performance of the algorithm. The specific formulae are as follows:

#### Metric 1: spacing (SP)

This indicator reflects how non-dominated solutions are distributed. The lower the value of SP, the more uniform the distribution of non-inferior solution sets will be.

11 $$ SP = \sqrt {\frac{{\sum\nolimits_{i = 1}^{np} {\left( {d_{i} - \overline{d} } \right)^{2} } }}{np}} $$

where $d_{i}$ is the minimum distance between two solutions. $\overline{d}$ is the average of all $d$. $np$ is the population size.

#### Metric 2: mean ideal distance (MID)

This indicator reflects the distance between the non-dominated solution and the ideal solution. A lower MID value means that the solution set is closer to the ideal solution.

12 $$ MID = \frac{{\sum\nolimits_{i = 1}^{np} {\sqrt {f_{1}^{2} + f_{2}^{2} } } }}{np} $$

where $f_{1}$ and $f_{2}$ are the values of objective function 1 and objective function 2, respectively.

As illustrated in Table 3, the performance of the algorithm is superior when the maximum generation is 4000.

**Table 3:** Performance of algorithms under different generations.

Generation	Spacing	Mean ideal distance
1000	0.224495	126.494
2000	0.149232	123.105
3000	0.169901	130.568
4000	0.131081	122.352

### Decision-making method

There are many schemes in the Pareto Front. The decision-makers need to decide on a preferred solution among options. The TOPSIS model can be adapted to evaluate the WRAS.

Construct weighted decision matrix:

13 $$ v_{ij} = w_{j} p_{ij} $$

14 $$ L = (v_{ij} )_{n \times m} $$

where $L$ is the weighted decision matrix, $w_{j}$ is the weight of objective function $j$, and $p_{ij}$ is the standardized value of objective function $j$ in scheme $i$.

Calculate the ideal solution:

15 $$ v_{j}^{ + } = max(v_{ij} ) $$

16 $$ v_{j}^{ - } = min(v_{ij} ) $$

where $v_{j}^{ + }$ and $v_{j}^{ - }$ respectively represent the ideal solution of positive objective and negative objective.

Calculate the Euclidean distance between each objective function value.

17 $$ V_{j}^{ + } = \sqrt {\sum\limits_{i = 1}^{k} {(v_{j}^{ + } - v_{ij} )^{2} } } $$

18 $$ V_{j}^{ - } = \sqrt {\sum\limits_{i = 1}^{k} {(v_{ij} - v_{j}^{ - } )^{2} } } $$

where $V_{j}^{ + }$ and $V_{j}^{ - }$ are the Euclidean distance.

Calculate the closeness degree. The value of closeness degree can represent the closeness degree between each scheme and the optimal scheme. The larger the value is, the better the regional state is.

19 $$ H_{j} = \frac{{V_{j}^{ - } }}{{V_{j}^{ + } + V_{j}^{ - } }} $$

where $H_{j}$ is the closeness value.

## Results

In this section, the calculation results of the model are comprehensively discussed. In this paper, three scenarios are presented to reflect socio-economic development trends. The practicability of the equilibrium theory-based allocation model is verified. The contradiction of water resources in the 7 cities is relieved.

### Water demand forecast

**Figure 3:**
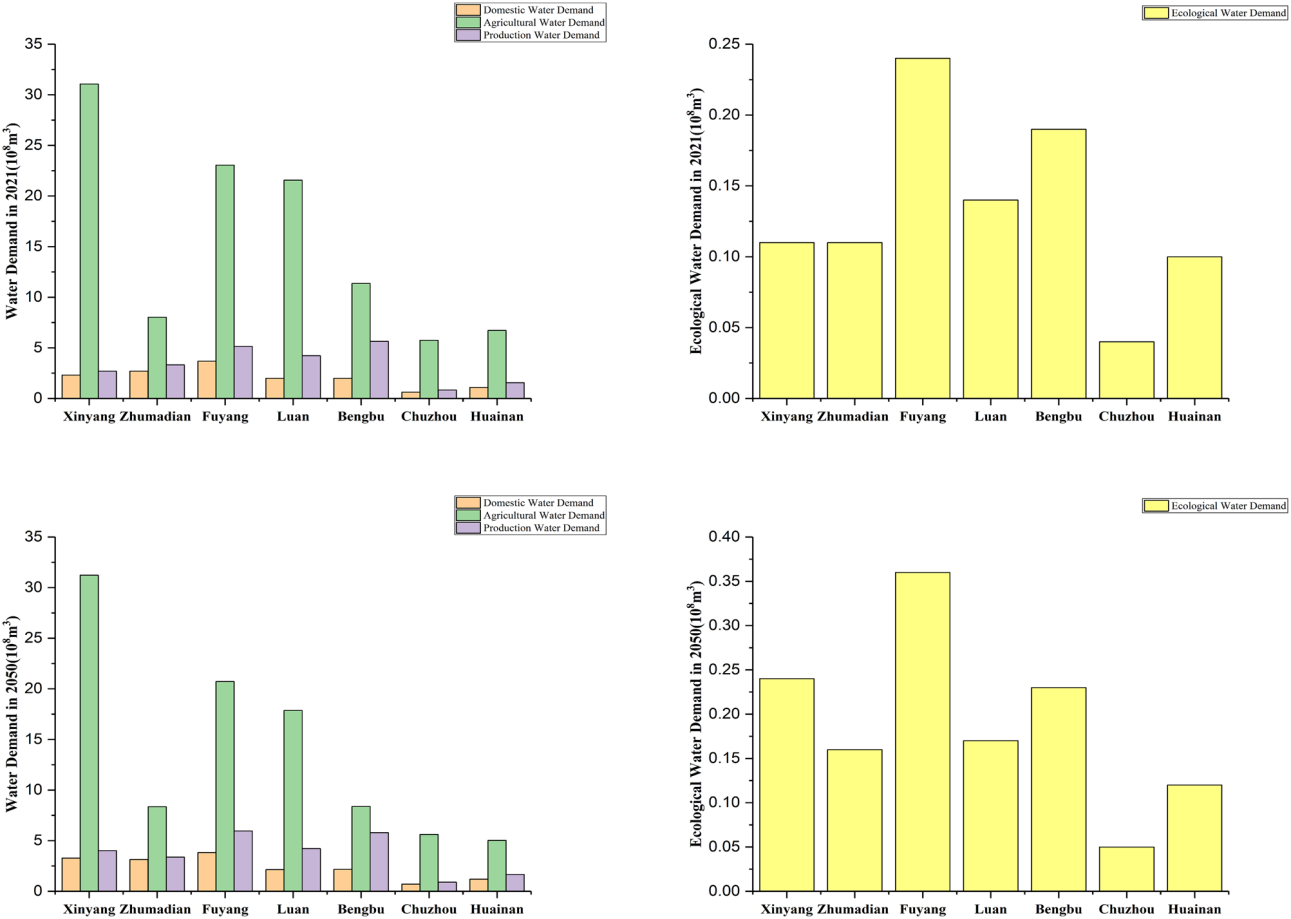
The water demand for each city in 2021 and 2050.

According to the actual water consumption situation and the future development plan of each sub-regions, combined with Quota method^2^, the water requirement of each water-using sector in 2021 and 2050 is shown in Fig. 3 and Table 4.

**Table 4:** Forecast of water demand in 2021 and 2050 (10^8^ m^3^).

	Domestic	Agricultural	Production	Ecological
2021	14.38 (9.83%)	107.53 (73.52%)	23.41 (16.01%)	0.93 (0.64%)
2050	17.46 (11.69%)	102.99 (68.96%)	38.74 (18.41%)	1.41 (0.94%)

From the perspective of the water-using sector, domestic water demand and production water demand have increased due to the economic development and the increase in population. Domestic water demand increased from 14.38 (10^8^ m^3^) in 2021 to 17.46 (10^8^ m^3^) in 2050. Production water demand increased from 23.41 (10^8^ m^3^) in 2021 to 38.74 (10^8^ m^3^) in 2050. As a result of the restructuring of agricultural cultivation, agricultural water demand decreases from 107.53 (10^8^ m^3^) in 2021 to 102.99 (10^8^ m^3^) in 2050. In general, the total water demand in the 2050 planning year is on an upward trend due to the growth in domestic water demand and production water demand.

### Pareto front characteristics

**Figure 4:**
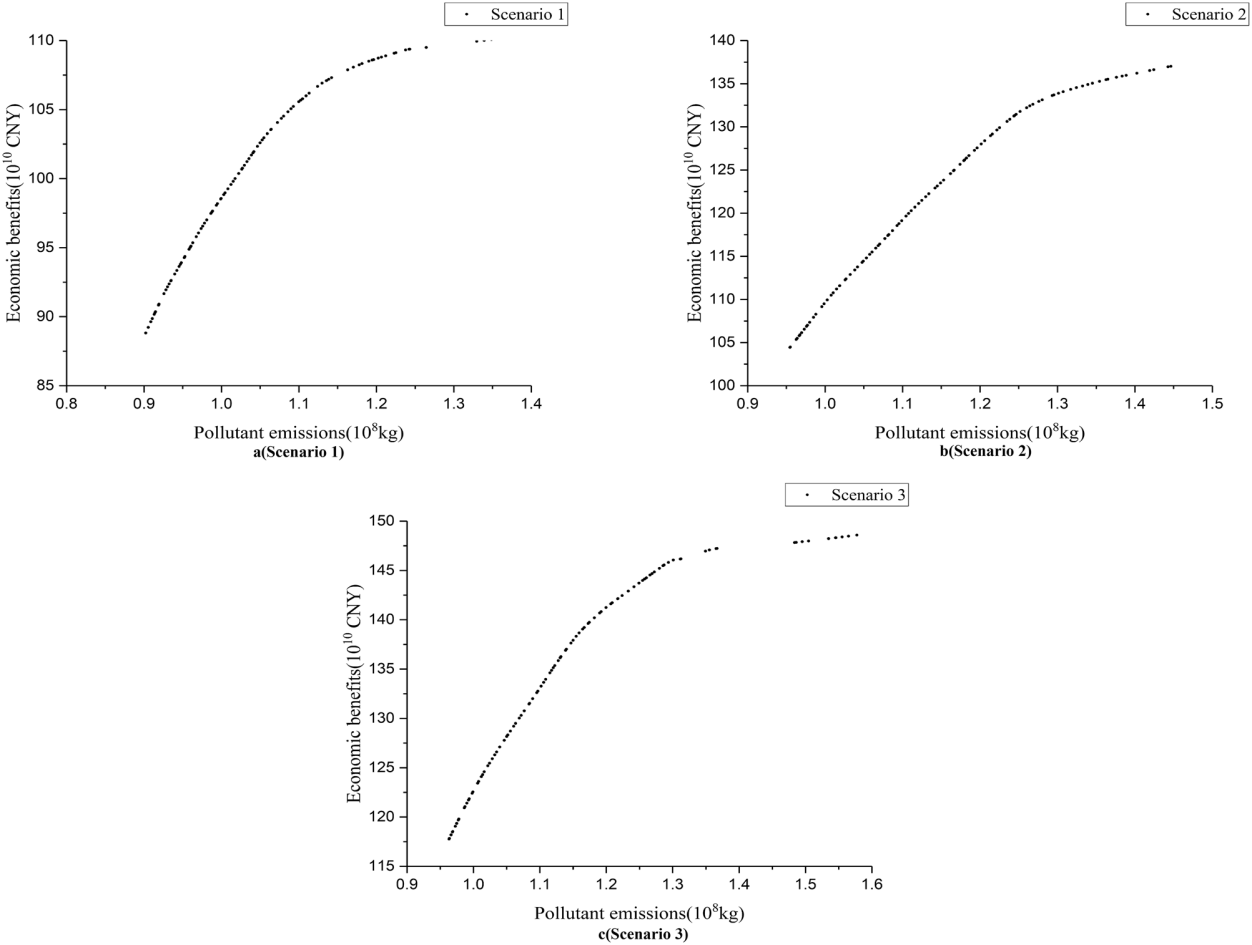
The Pareto frontier of water resources allocation.

Figure 4 illustrates the Pareto front between the two objective functions. The lower the pollutant emissions indicates a better sustainability of the WRA. A higher economic efficiency implies a more efficient allocation of water resources. Consequently, higher sustainability objective corresponds to poorer economic performance. In addition to this, pollutant emissions increase with economic benefits, translating into a poorer sustainability. The two objective functions demonstrate the conflict between sustainability and economic efficiency in water allocation. The basin authorities need to make trade-offs between the two objective functions to identify appropriate allocation schemes. If the basin authority expects to achieve maximum economic benefits, the scheme on the right in Fig. 4 will be chosen. If the basin authority prefers to achieve maximum sustainability, the option on the left in Fig. 4 will be followed.

### Total economic benefits analysis

**Figure 5:**
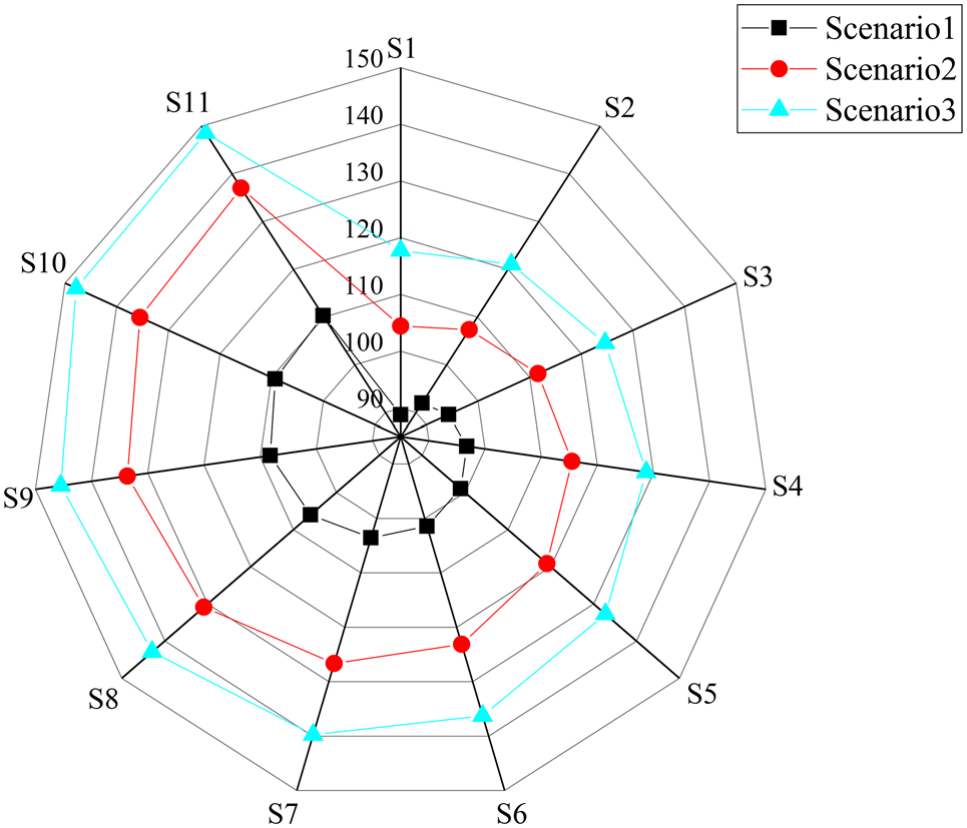
The economic benefits of WRA under three scenarios (10^10^ CNY).

11 Pareto sets of efficiency and sustainability objectives with a uniform distribution are applied to analyze the economic benefits of the 11 scenarios. Figure 5 shows the total GDP brought by WRA to the whole study area. Overall, the economic benefits will improve over time. The average economic benefits for Scenarios 1, 2 and 3 are 100.94 (10^10^CNY), 122.31 (10^10^CNY) and 135.14 (10^10^CNY), respectively. In general, if the basin authorities are willing to discharge more pollutants, there will be a higher economic benefit to the basin. As a result, basin authorities have to make decisions between economic efficiency and pollutant discharge, provided that pollutant discharge limits are met.

### Water deficit rate analysis

**Figure 6:**
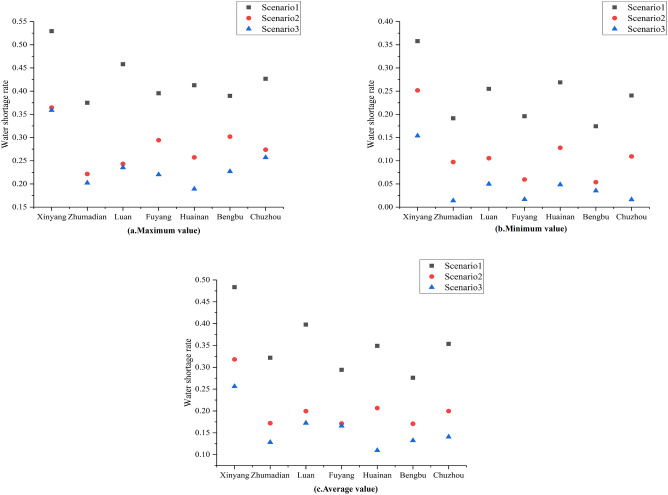
Water deficit rates for each city under three scenarios.

Figure 6 demonstrates the maximum, minimum and average values of water deficit rate in each region for the three scenarios. In terms of the study area, with the restructuring of the industrial and agricultural cultivation structure, the water shortage rate for Scenario 2 is lower than that of Scenario 1. With improved water use efficiency, Scenario 3 has the lowest water shortage rate, with the overall average water shortage rate in the study area decreasing from 38.12 to 18.11%.

For specific cities, no matter what the decision-making preferences for basin management are, Xinyang and Luan have extremely large water resources deficits, with maximum deficits of 45% or more. With the modification of the agricultural cultivation structure, the maximum water deficit rate could be reduced to 35.77% and 25.49% in Xinyang and Luan respectively. The potential reasons for this are the extremely large agricultural water demands of Xinyang and Luan. Hence, basin management authorities need to balance water demand between water-using sectors. In addition to this, the management authorities need to optimize the cropping structure and promote agricultural water-saving techniques.

### Equilibrium analysis

11 uniformly distributed Pareto sets of efficiency and sustainability objectives were adopted to analyze the correlation between the Gini coefficient and economic benefits. Figures 5 and 7 demonstrate that the Gini coefficient for schemes S1-S11 increases with the upward economic efficiency and that there is a positive correlation between the Gini coefficient and economic efficiency as well. Consequently, increasing economic efficiency may lead to a poorer regional equilibrium in the WRA.

**Figure 7:**
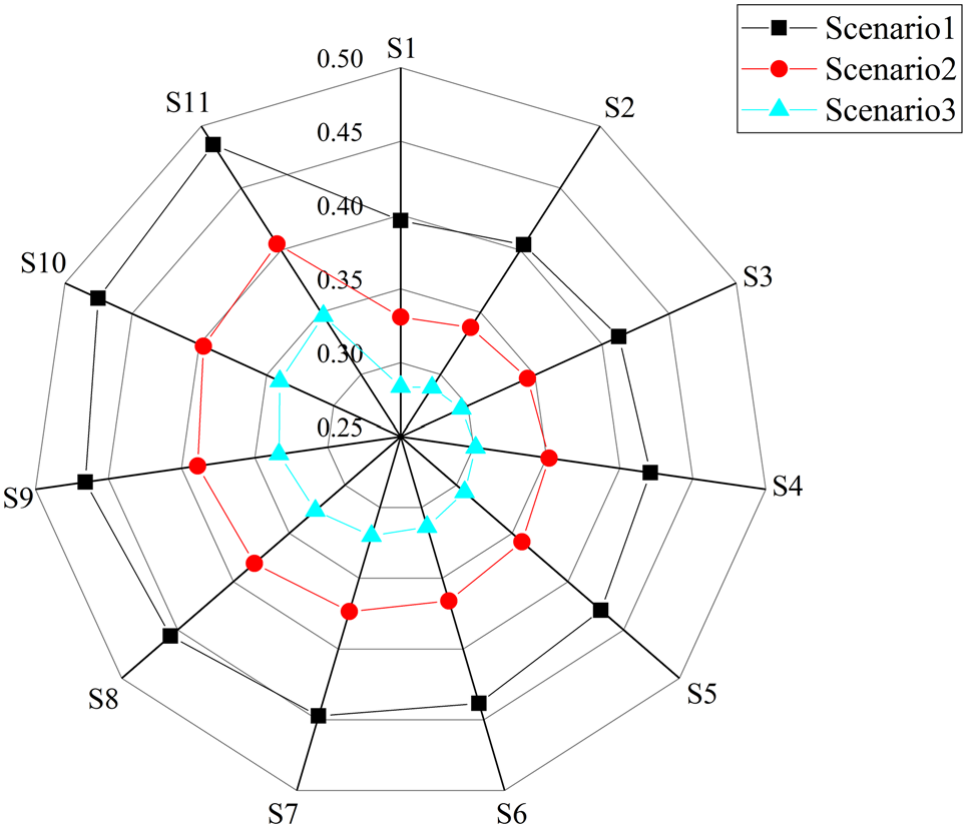
Gini coefficient for the study area under the three scenarios.

Based on a previous study^25^, when the Gini coefficient is less than 0.3, the distribution has higher equity. When the Gini coefficient is less than 0.4, the distribution is more reasonable. Figure 7 illustrates that the average value of the Gini coefficients for Scenarios 1, 2 and 3 are 0.439, 0.367 and 0.314, respectively. It is evident that the WRA in the 2021 planning year is in an unbalanced state. With industrial restructuring, the WRA in the 2050 could reach a barely equilibrium state. The WRA scheme for the study area can attain a state of regional equilibrium under the scenario of a 10% improvement in water use efficiency.

In addition, the socio-economic and eco-environmental subsystems have a coupling coordination degree of 0.7, and the study area reaches system equilibrium. Meanwhile, the basin authorities can make decisions on schemes based on pollutant discharge limits.

### Decision-making analysis

Table 5 shows the maximum and minimum values of the two objective functions for the three scenarios. It can reflect the conflicting relationship between the two objectives, where the maximum economic benefit objective is matched with the minimum sustainability objective. Scheme B is preferred under the three scenarios when the basin authorities strive for maximum economic efficiency. When the management pursues the maximum sustainable development, Scheme A will stand out.

**Table 5:** The maximum and minimum values of the objective function for the three scenarios.

Scenario	Scheme	$f_{1}$	$f_{2}$
Scenario1	Scheme A (min.value of $f_{2}$)	88.817	0.902
	Scheme B (max.value of $f_{1}$)	110.288	1.379
Scenario2	Scheme A (min.value of $f_{2}$)	104.441	0.954
	Scheme B (max.value of $f_{1}$)	137.016	1.446
Scenario3	Scheme A (min.value of $f_{2}$)	117.761	0.963
	Scheme B (max.value of $f_{1}$)	148.579	1.57

However, neither Scheme A nor Scheme B integrates the efficiency and sustainability of WRA. Therefore, this study applies the TOPSIS model to seek better decision results. 11 representative schemes are selected uniformly according to the Pareto frontier. S1 and S11 represent the two extreme schemes. Based on the TOPSIS model, we can calculate the relative closeness of the ideal solution for each scheme. The weights are set as the preference of the solution set to the objective function.

**Figure 8:**
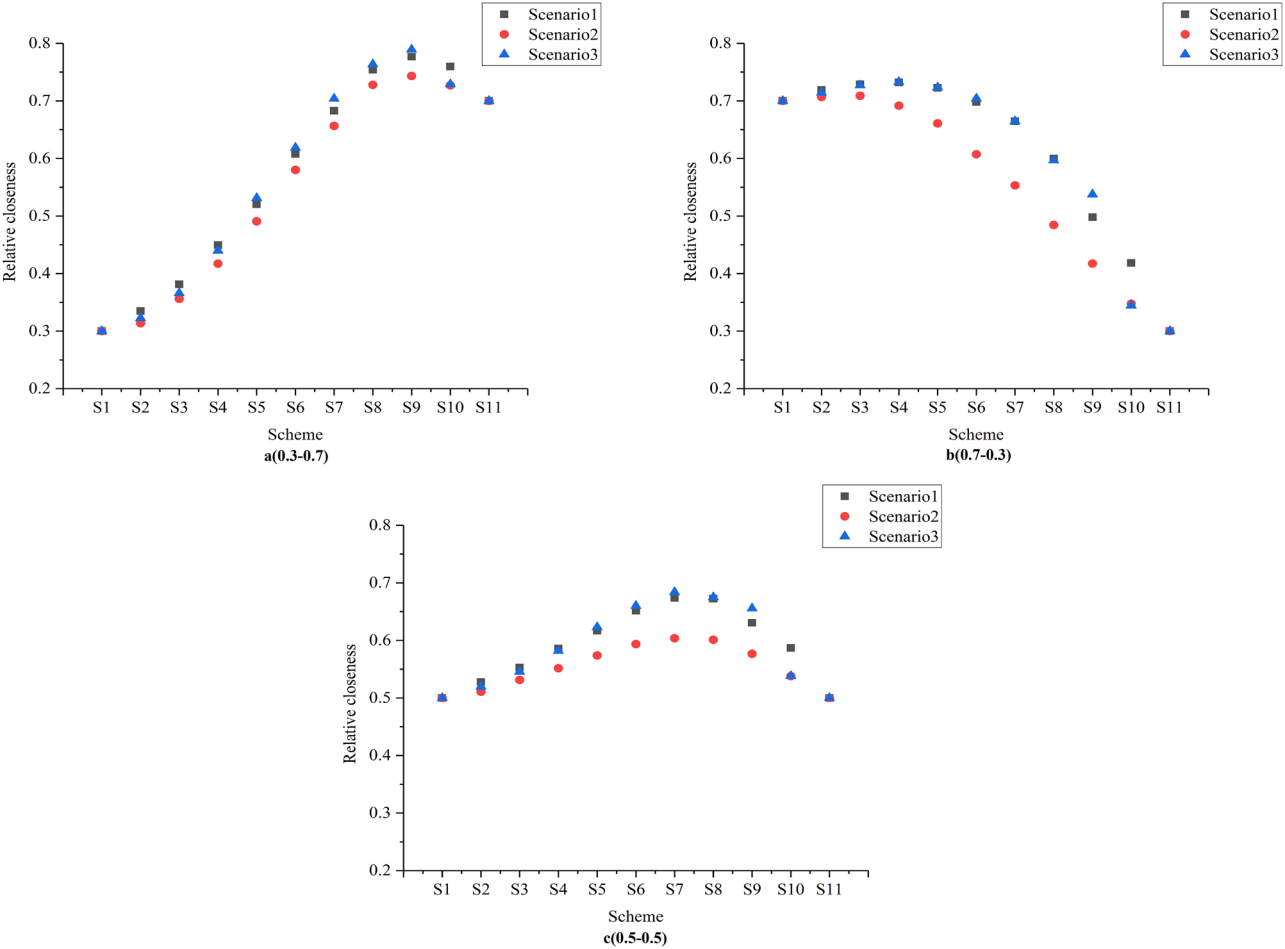
Relative posting values for the three weighting sets (0.3–0.7 means that the weights of objective function one and objective function two are 0.3 and 0.7 respectively).

The relative closeness values for the weight settings are shown in Fig. 8. Relative closeness allows decision makers to make decisions more efficiently. As demonstrated in Fig. 8, if the decision-makers consider economic efficiency and sustainability to be equally important, they can choose scheme S7. Hence, the TOPSIS model allows for a quantitative evaluation of the non-inferior solution set so that the decision-makers can make a decision.

## Conclusions

To establish a WRAM that can maintain social harmony and stability, and maintain equity and efficiency, this study illustrates a WRAM based on equilibrium theory. In addition, taking 7 cities in the Huaihe River Basin as the study area, this study finds that the WRAS shows that the optimal allocation model based on equilibrium theory can not only realize the efficient and equitable water resources distribution but also realize a balance between different sub-regions and a balance between different systems, providing a variety of water resources allocation schemes for the basin management department. The main conclusions are as follows:

1. In the planning year 2050, the total water demand in the middle and upper reaches of the Huaihe River Basin at 75% inflow frequency is 160.6 billion m^3^, a slight increase from 2021.
2. Economic efficiency and environmental sustainability present conflicting relationships in WRA. Pollutant emissions will increase with economic benefits increasing.
3. With the adjustment of industrial structure and the improvement of water resources utilisation efficiency, the conflict between supply and demand in the study area has been alleviated and economic benefits has been improved.
4. Decision-makers can apply the TOPSIS model to evaluate and make decisions on the solution. If the decision-makers consider that both objective weights are equally important, they can choose scheme S7, which has the largest relative closeness value.

This study could be applied to basin water resources management and could be considered as an approach for basin management to achieve balanced regional development and balanced system development.
